# Supplementary material for: Isolated tooth reveals hidden spinosaurid dinosaur diversity in the British Wealden Supergroup (Lower Cretaceous)
Source: PeerJ. 2023 May 31;11:e15453. doi: 10.7717/peerj.15453 (PMC10239232; doi:10.7717/peerj.15453)

Supplementary information: Isolated teeth reveal hidden spinosaurid dinosaur diversity in the British Wealden Supergroup (Lower Cretaceous)

Chris Tijani Barker, Darren Naish, Neil J. Gostling

1. Analysis of HASMG G369a

1.1. Full measurements of HASMG G369a

Table S1. Measurements in millimetres (mm). Values based on reconstructed crown height (bold; see main text) marked with an asterisk (*).

Crown base length (CBL)	8.16
Crown base width (CBW)	7.03
**Crown height (CH)**	**17.2**
Apical length (AL)	?
Crown angle (CA)	74
Crown base ratio (CBR)	0.86
Crown height ratio (CHR)	2.1*
Midcrown length (MCL)	5.67*
Midcrown width (MCW)	4.54*
Midcrown ratio (MCR)	0.8*
Mesiobasal Denticle Extension (MDE)	?
Mesiobasal Carina Extension (MCE)	?
Mesial Serrated Carina Length (MSL)	?
Distal Serrated Carina Length (DSL)	?
Mesial Carina Length (MCAL)	?
Distal Carina Length (DCAL)	?
Distal Denticle Height (DDH)	0.195*
Distal Denticle Length (DDL)	0.171*
Distal Denticle Width (DDW)	0.253*
Mesial Denticle Height (MDH)	?

Mesial Denticle Length (MDL)	?
Mesial Denticle Width (MDW)	?
Distal Denticle Height Ratio (DHR)	1.14*
Distal Denticle Base Ratio (DBR)	1.48*
Mesial Denticle Height Ratio (MHR)	?
Mesial Denticle Base Ratio (MBR)	?
Distoapical Denticle Density (DA)	?
Distocentral Denticle Density (DC)	35*
Distobasal Denticle Density (DB)	?
Mesioapical Denticle Density (MA)	?
Mesiocentral Denticle Density (MC)	35*
Mesiobasal Denticle Density (MB)	?
Average Mesial Denticle Density (MAVG)	?
Average Distal Denticle Density (DAVG)	?
Denticle Size Density Index (DSDI)	1*
Transverse Undulation Density (TUD)	n/a
Marginal Undulation Density (TUD)	n/a
Labial Flutes (LAF)	7
Lingual Flutes (LAF)	5

1.2. Regression analyses

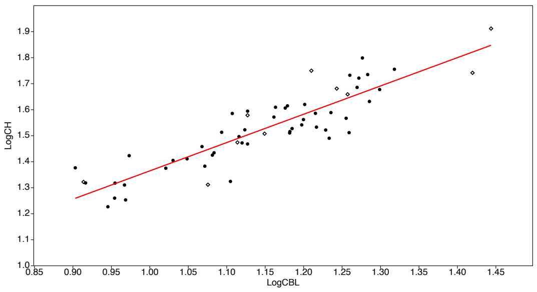

All spinosaurids (all positions), LogCH vs. LogCBL.
Slope: 1.0881, **intercept**: 0.27671, r2: 0.79069.

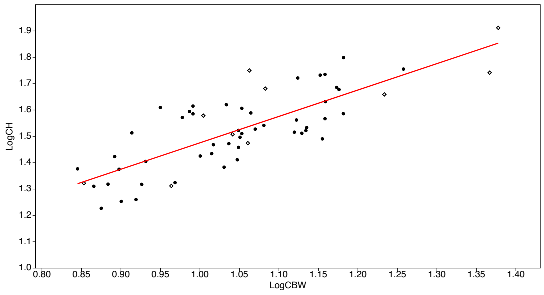

All spinosaurids (all positions), LogCH vs LogCBW.
Slope: 1.002, **intercept**: 0.47343, **r2**: 0.62588.

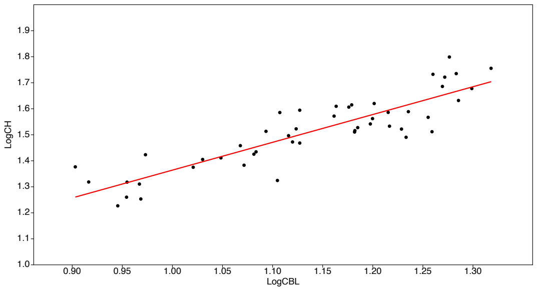

Baryonychine spinosaurids (all positions), LogCH vs. LogCBL.
Slope: 1.0686, **intercept**: 0.29548, **r2**: 0.77314.

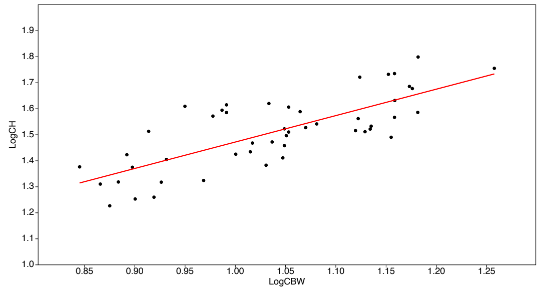

Baryonychine spinosaurids (all positions), LogCH vs. LogCBW.
Slope: 1.0161, **intercept**: 0.45603, **r2**: 0.582.

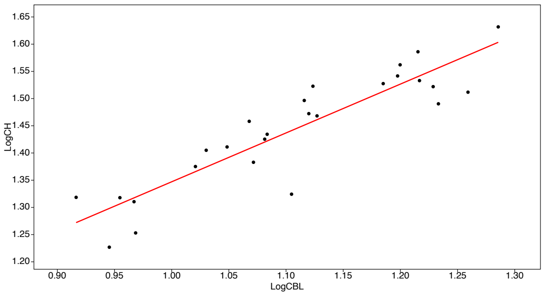

***Baryonyx* (all positions), LogCH vs. LogCBL.**
Slope: 0.89772, **intercept**: 0.44935, **r2**: 0.81304.

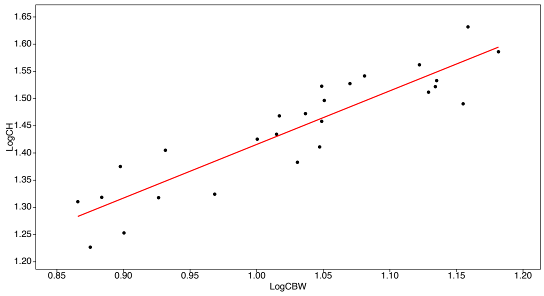

***Baryonyx* (all positions), LogCH vs. LogCBW.**
Slope: 0.9852, **intercept**: 0.43051, **r2**: 0.8198.

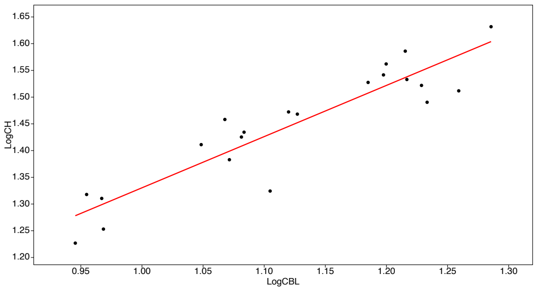

***Baryonyx* (lateral teeth only), LogCH vs. LogCBL.**
Slope: 0.95799, **intercept**: 0.37236, **r2**: 0.83866.

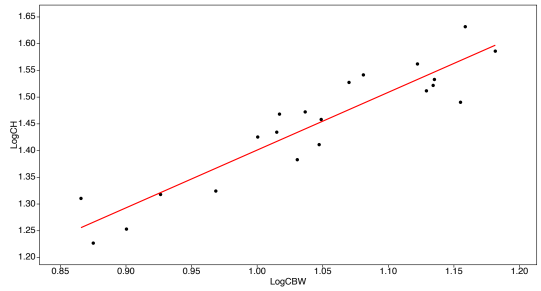

***Baryonyx* (lateral teeth only), LogCH vs. LogCBW.**
Slope: 1.0807, **intercept**: 0.32022, **r2**: 0.86066.

1.3. Full phylogenetic results

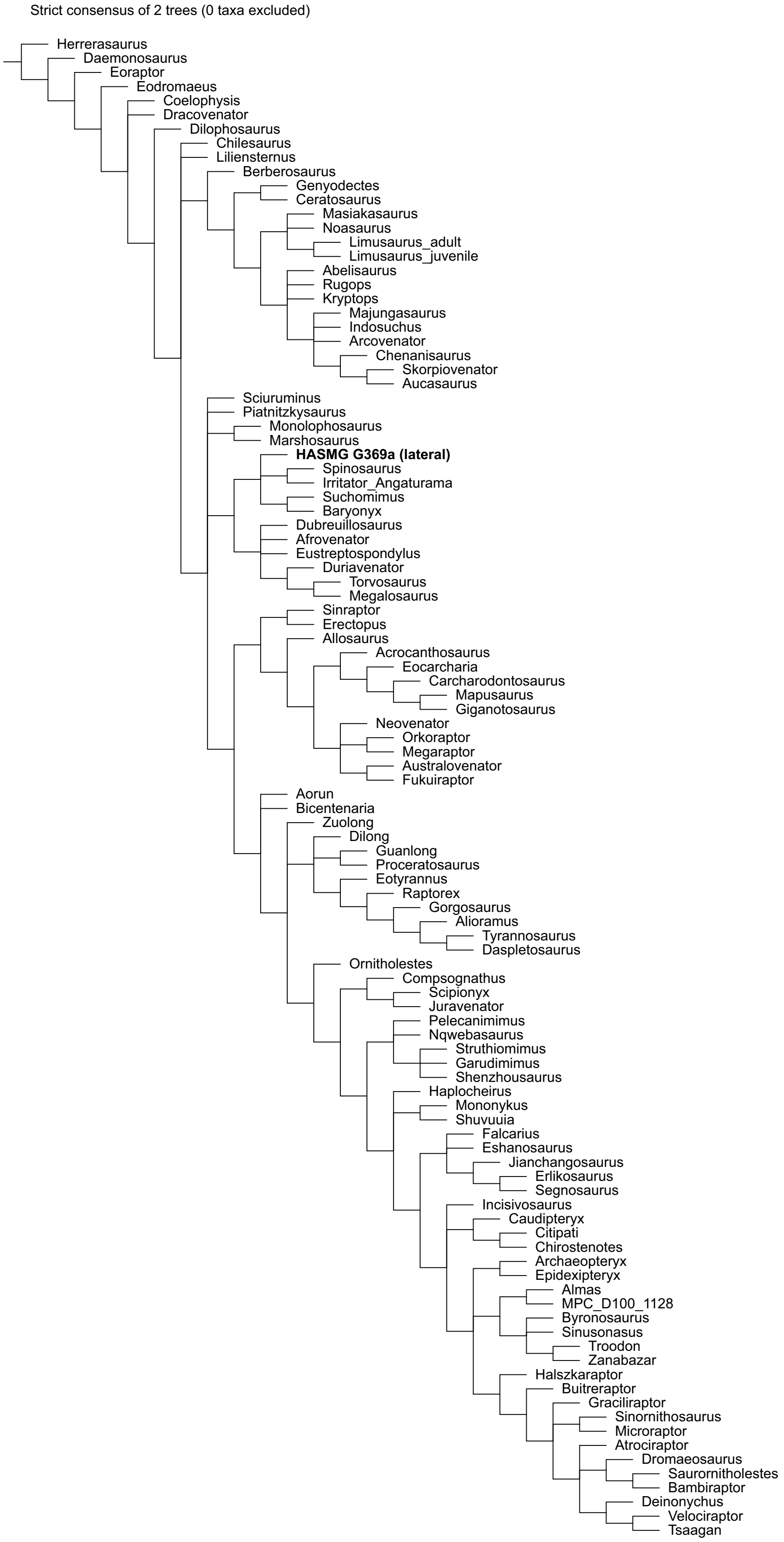

Fig S1 Strict consensus of the constrained analysis using the whole dentition dataset.

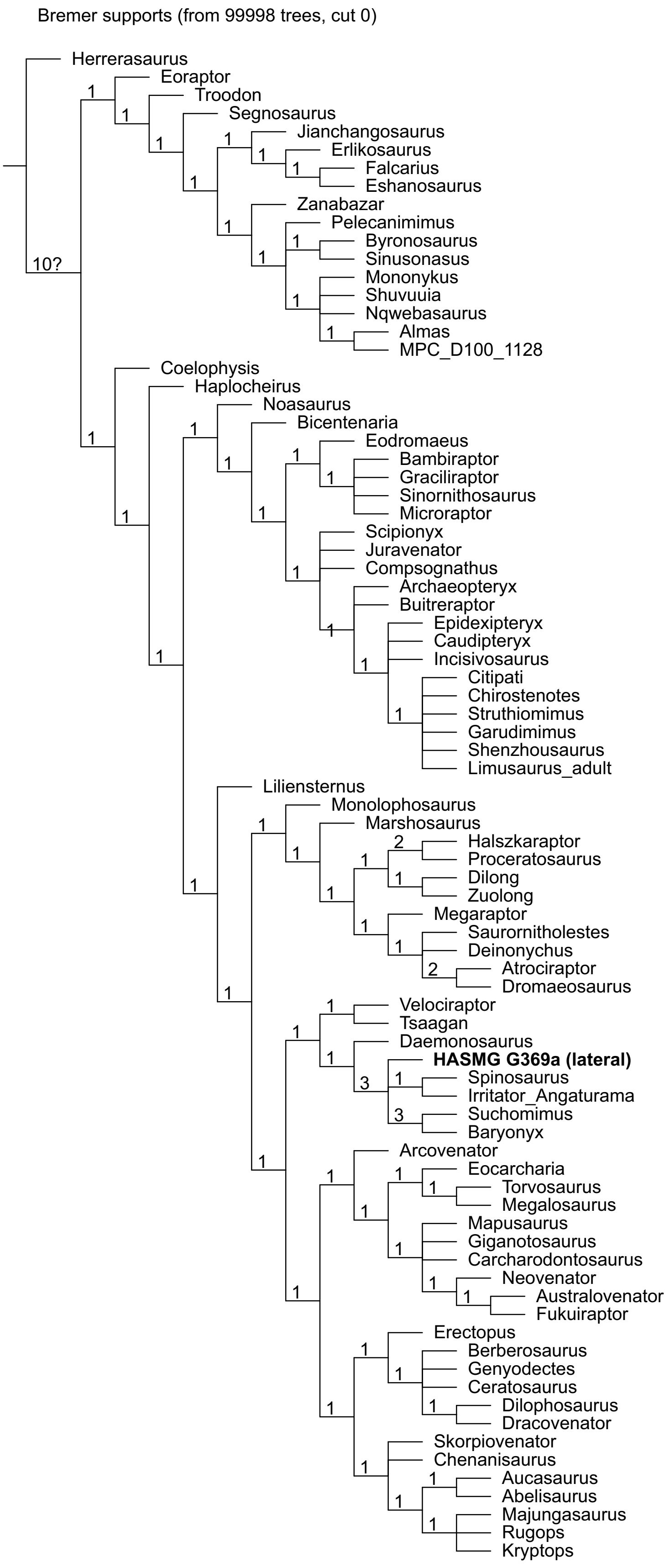

Fig S2 Reduced consensus of the unconstrained analysis using the whole dataset.

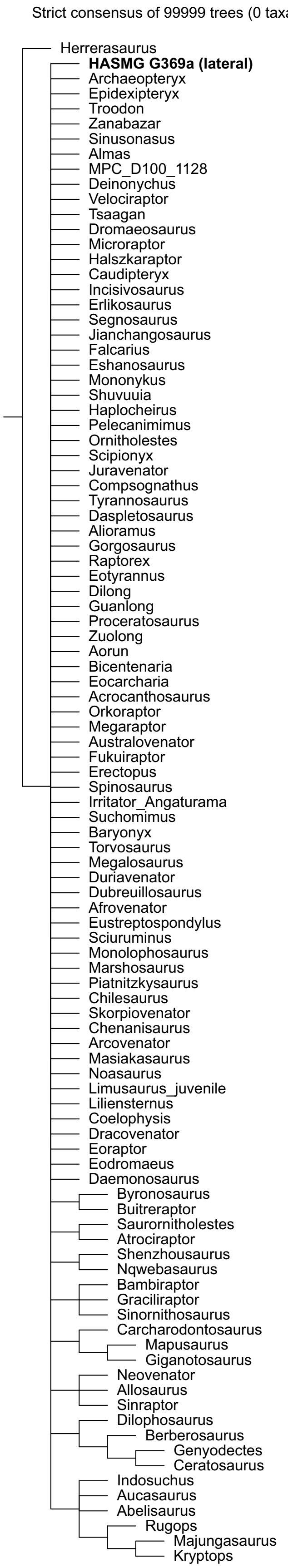

Fig S3 Strict consensus of the unconstrained analysis using the crown-only dataset.

1.4. Cluster analyses:

Whole dataset – Hierarchical clustering

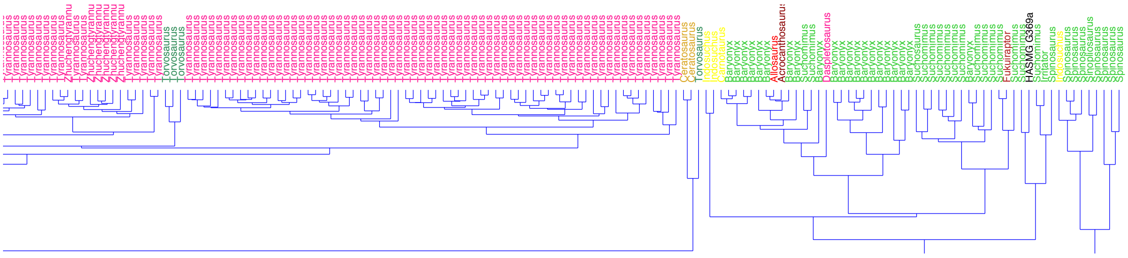

Whole dataset (no denticles = ?) – Hierarchical clustering

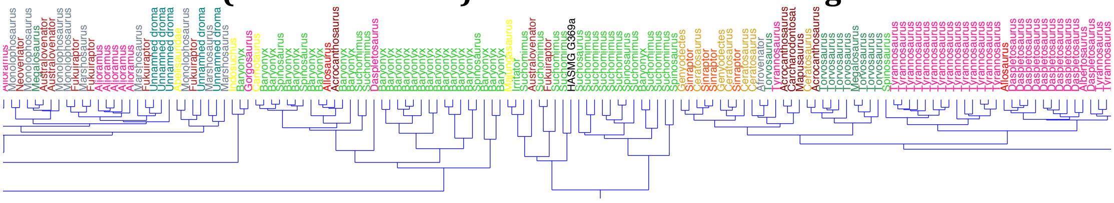

Reduced dataset – Hierarchical clustering

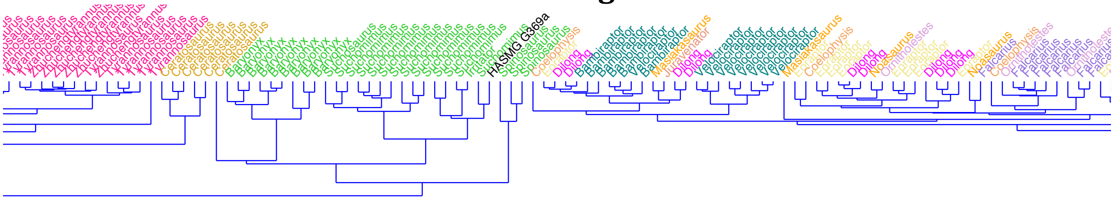

Reduced dataset (no denticles = ?) – Hierarchical clustering

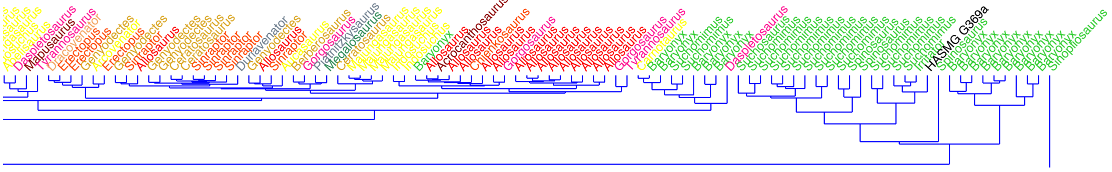

Whole dataset – Neighbour-joining

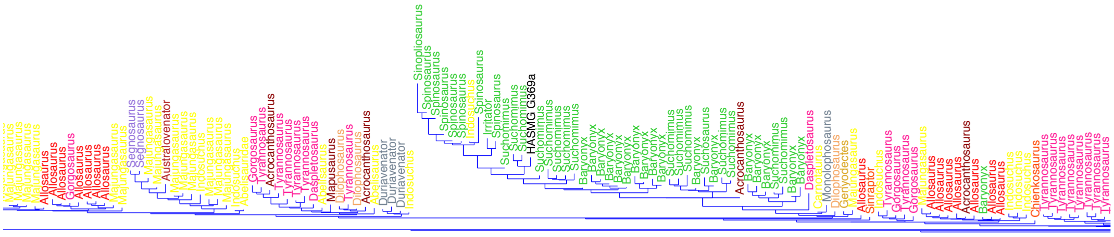

Whole dataset (no denticles = ?) – Neighbour-joining

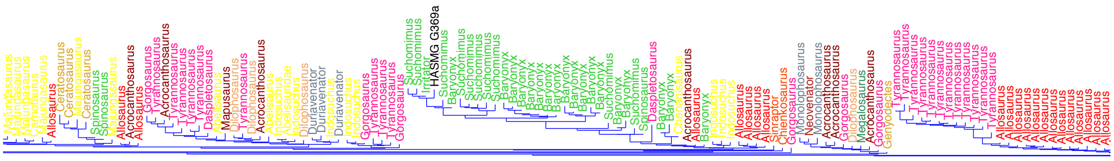

Reduced dataset – Neighbour-joining

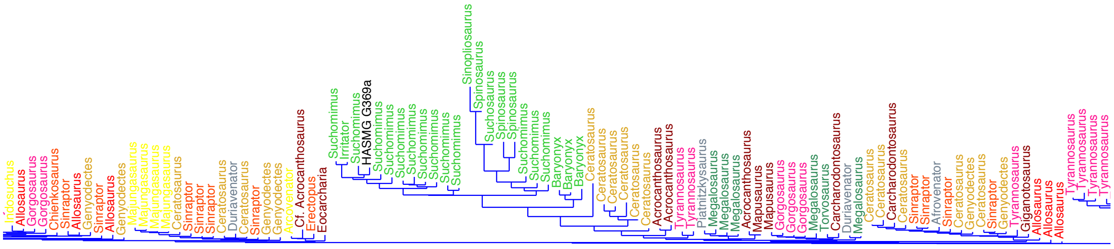

Reduced dataset (no denticles = ?) – Neighbour-joining

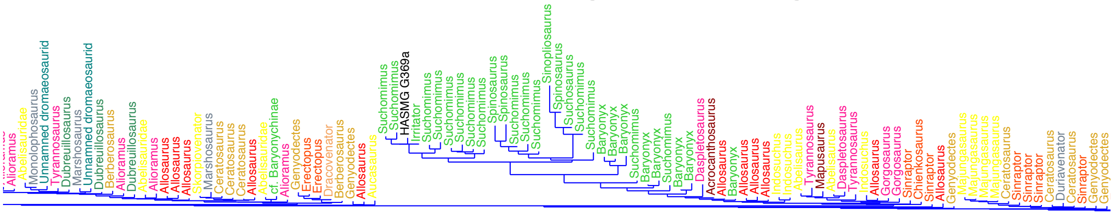

2. Quantitative analysis of XMDFEC V10010

2.1. Discriminant function analyses: results

Table S2. Results of the discriminant function analyses on the various iterations of the pan-theropodan dataset, with XMDFEC V10010 treated as an unknown taxon

**Dataset**	**Discriminant Function Analysis**		**Reclassification Rate (RR)**	
	**Clade level**	**Taxon level**	**Clade level (%)**	**Taxon level (%)**
Whole dataset	Metriacanthosauridae	*Skorpiovenator*	60.84	61.14
Whole dataset (no denticles = ?)	Metriacanthosauridae	*Condorraptor*	62.12	59.34
My dataset	Metriacanthosauridae	*Skorpiovenator*	54.46	63.68
My dataset (no denticles = ?)	Metriacanthosauridae	*Erectopus*	59.29	60.3
Whole dataset with large teeth	Allosauridae	*Erectopus*	59.2	57.4
Whole dataset with large teeth (no denticles = ?)	Allosauridae	*Erectopus*	62.1	57.54
My dataset with large teeth	Metriacanthosauridae	*Erectopus*	58.54	61.56
My dataset with large teeth (no denticles = ?)	Metriacanthosauridae	*Erectopus*	60.8	62.06

**Clade level**	**Taxon level**	**Clade level (Eigenvalue)**	**Taxon level (Eigenvalue)**

PC1 (%)	PC2 (%)	PC1 (%)	PC2 (%)	Axis 1	Axis 2	Axis 1	Axis 2
50.89	19.86	41.04	21.57	5.7081	2.2272	18.374	9.6563
50.08	19.11	42.86	17.09	5.7905	2.2091	18.01	7.1806
57.04	21.78	41.07	24.73	12.239	4.6732	24.992	15.051
54.09	22.79	41.4	25.68	11.008	4.6388	23.75	14.731
37.97	31.01	35.48	27.85	1.9237	1.5713	6.2636	4.9158
39.64	30.5	36.25	22.59	2.2061	1.6975	6.0601	3.7752
48.45	27.08	54.31	18.43	2.2474	1.2559	12.99	4.4081
57.88	22.98	45.27	21.19	3.4446	1.3678	9.3725	4.3866

2.2. Cluster analyses: results

Table S3. Results of the cluster analyses on the various iterations of the pan-theropodan datasets, with XMDFEC V10010 treated as an unknown taxon.

Dataset	Cluster Analysis	
	Hierarchical clustering	Neighbour joining
Whole dataset	Abelisauridae indet.	Abelisauridae indet. + *Fukuiraptor*
Whole dataset (no denticles = ?)	Abelisauridae indet.	Abelisauridae indet. + *Fukuiraptor*
My dataset	Abelisauridae indet.	*Arcovenator*
My dataset (no denticles = ?)	Abelisauridae indet.	Abelisauridae indet.
Whole dataset with large teeth	Abelisauridae indet.	Abelisauridae indet.
Whole dataset with large teeth (no denticles = ?)	Abelisauridae indet.	Abelisauridae indet. + *Fukuiraptor*
My dataset with large teeth	Abelisauridae indet.	Abelisauridae indet.
My dataset with large teeth (no denticles = ?)	Abelisauridae indet.	Abelisauridae indet.

Whole dataset – Hierarchical clustering

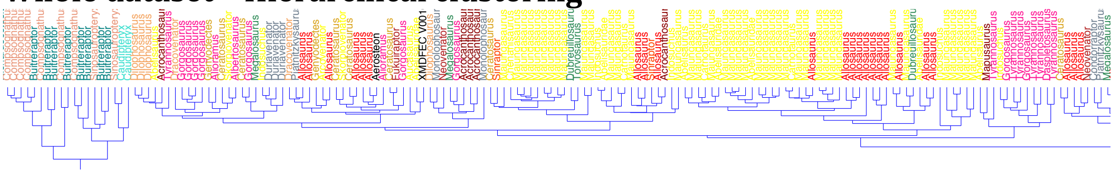

Whole dataset (no denticles = ?) – Hierarchical clustering

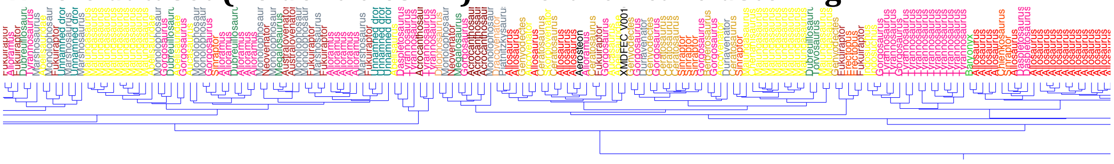

Reduced dataset – Hierarchical clustering

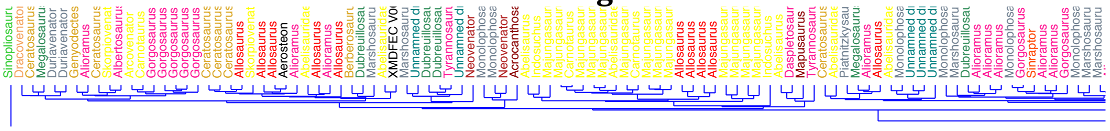

Reduced dataset (no denticles = ?) – Hierarchical clustering

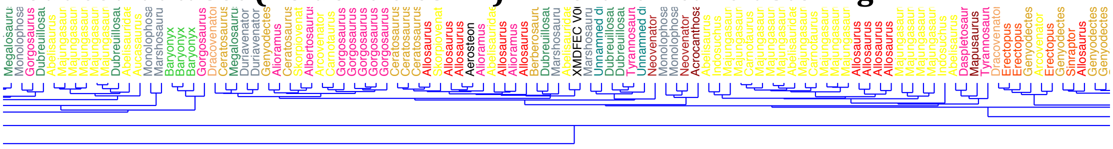

Whole dataset large teeth only – Hierarchical clustering

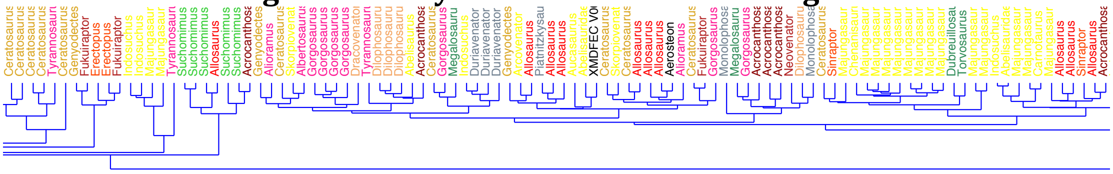

Whole dataset large teeth only (no denticles = ?) – Hierarchical clustering

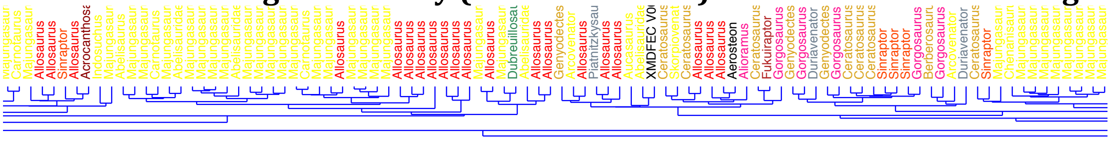

Reduced dataset with large teeth – Hierarchical clustering

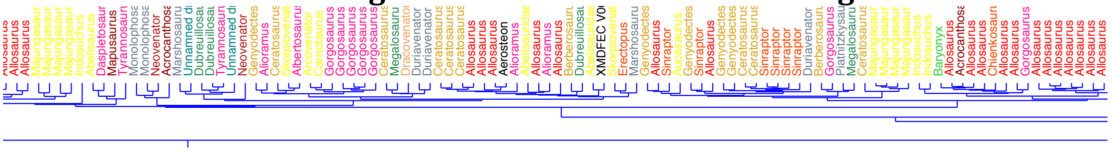

Reduced dataset with large teeth (no denticles = ?) – Hierarchical clustering

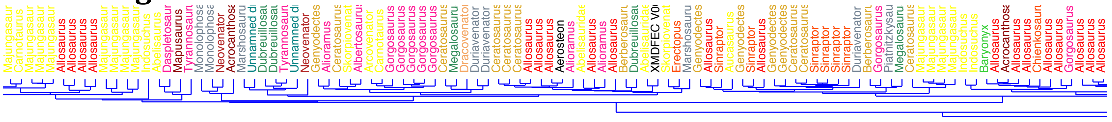

Whole dataset – Neighbour-joining

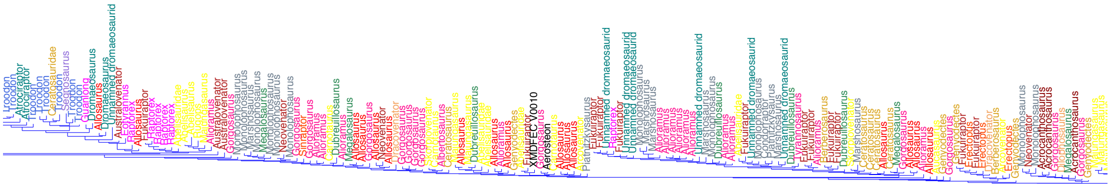

Whole dataset (no denticles = ?) – Neighbour-joining

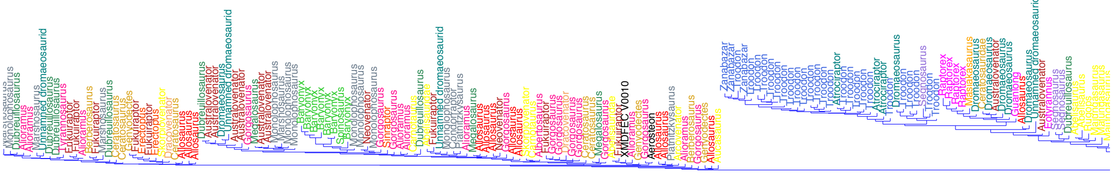

Reduced dataset – Neighbour-joining

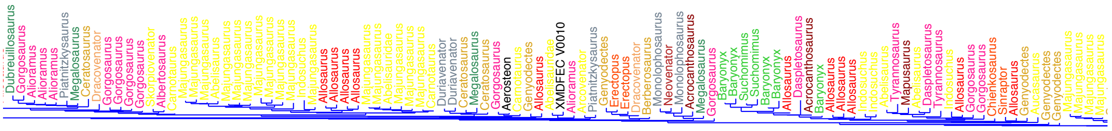

Reduced dataset (no denticles = ?) – Neighbour-joining

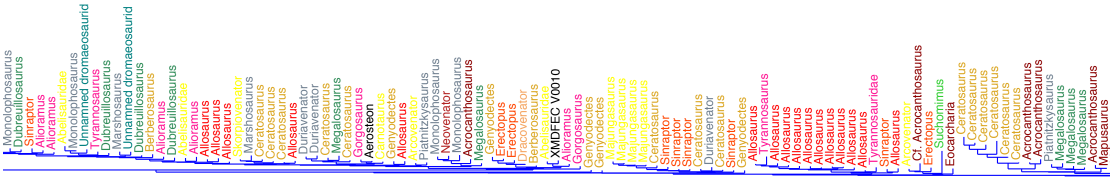

Whole dataset with large teeth– Neighbour-joining

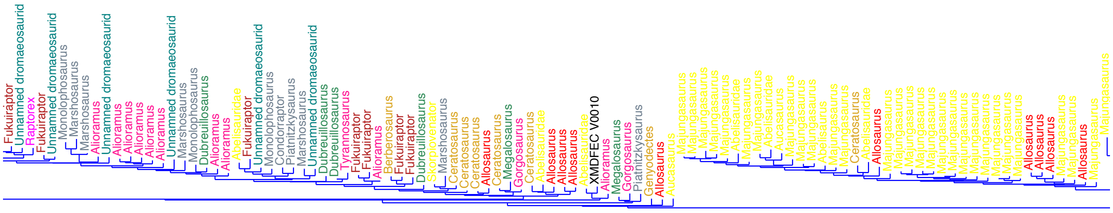

Whole dataset with large teeth (no denticles = ?) – Neighbour-joining

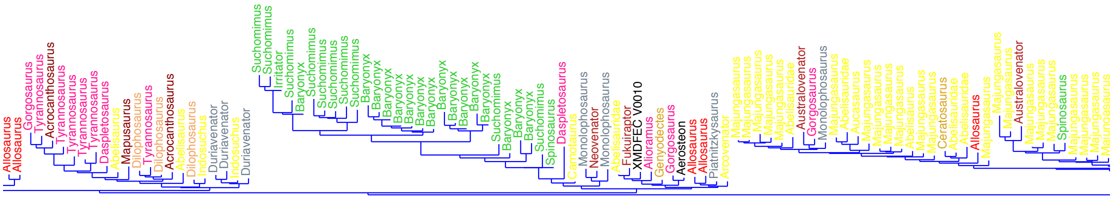

Reduced dataset with large teeth – Neighbour-joining

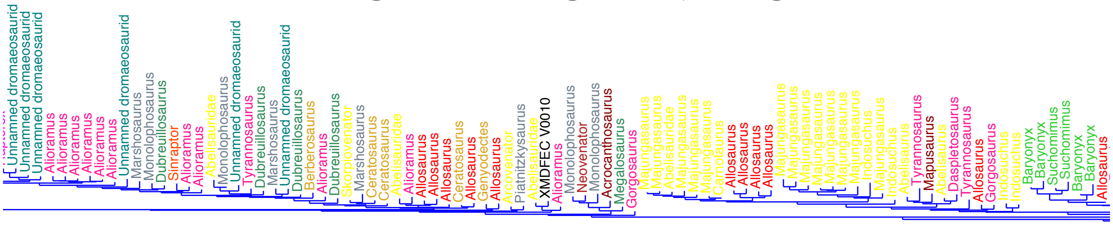

Reduced dataset with large teeth (no denticles = ?) – Neighbour-joining

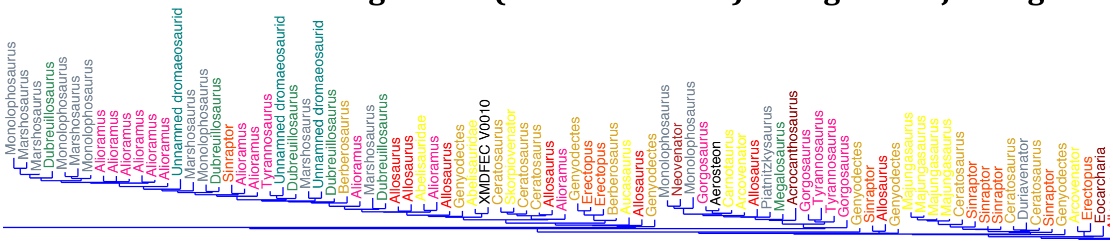

Supplement: Supplemental Information 2 — (1) Complete measurements of HASMG G369a, (2) results of the regression analyses used to estimate HASMG G369a crown height, (3) full phylogenetic results for the analyses involving HASMG G369a, (4) graphical representation of the cluster analyses used to classify HASMG G369a, and the full quantitative results (DFA and cluster) of the analysis of isolated tooth XMDFEC V10010. [file peerj-11-15453-s002.pdf]
